# A CLOSER LOOK AT OLDER ADULTS AS PEER EDUCATORS IN LIFELONG LEARNING INSTITUTIONS IN THE US

**DOI:** 10.1093/geroni/igad104.1746

**Published:** 2023-12-21

**Authors:** Giuliana Casanova, Joyce Weil, Margarida Cerqueira

**Affiliations:** University of Aveiro, Aveiro, Aveiro, Portugal; Towson University, Towson, Maryland, United States; Universidade de Aveiro, Aveiro, Aveiro, Portugal

## Abstract

Productive engagement in later life refers to paid or unpaid activities that can provide beneficial outcomes for individuals and society. This qualitative study examines the experiences of 17 peer educators, aged 61 to 84 and without cognitive deficits, who teach at three institutions of lifelong learning in Florida, USA. Data were collected through field notes, document analysis, a sociodemographic questionnaire, individual in-depth interviews, and brief interviews with each institution′s director. Descriptive statistics were used to examine the profiles of the peer educators, and emerging themes were identified through thematic analysis of the interview data. Preliminary results suggest that volunteerism and altruism played a role in their decision to become peer educators. Descriptive statistics showed that, on average, peer educators have obtained higher education degrees and have past teaching experience. The study identified several emerging themes related to the experiences of peer educators, including the need for purpose, the value of social connections, enjoyment of their role, and the importance of mutual learning. The analysis also revealed some challenges, including the need for environmental modifications to improve teaching conditions and assistance with marketing to promote classes and increase attendance. Overall, this study provides preliminary evidence to aid in understanding older adults in productive roles and fighting ageism. Identify current gaps in each institution′s approach, capacity to promote their participation and enhance their teaching experience while highlighting their contributions to society.

